# The microbial metabolic activity on carbohydrates and polymers impact the biodegradability of landfilled solid waste

**DOI:** 10.1007/s10532-021-09967-6

**Published:** 2021-11-23

**Authors:** Christian Brandstaetter, Nora Fricko, Mohammad J. Rahimi, Johann Fellner, Wolfgang Ecker-Lala, Irina S. Druzhinina

**Affiliations:** 1 Research Unit Waste and Resource Management, Institute for Water Quality and Resource Management, TU Wien, Karlsplatz 13/226.2, 1040 Vienna, Austria; 2 Institute of Computer Science, University of Applied Sciences Wiener Neustadt, Johannes-Gutenberg-Straße 3, 2700 Wiener Neustadt, Austria; 3 Institute of Chemical, Environmental and Bioscience Engineering (ICEBE), TU Wien, Gumpendorferstrasse 1a, 1060 Vienna, Austria; 4 Key Laboratory of Plant Immunity, Fungal Genomics Laboratory (FungiG), Nanjing Agricultural University, Weigang No. 1, Nanjing, 210095 People’s Republic of China

**Keywords:** Biodegradation, Biolog® EcoPlates™, Municipal solid waste, Gompertz equation, In-situ aeration

## Abstract

Biological waste degradation is the main driving factor for landfill emissions. In a 2-year laboratory experiment simulating different landfill in-situ aeration scenarios, the microbial degradation of solid waste under different oxygen conditions (treatments) was investigated. Nine landfill simulation reactors were operated in triplicates under three distinct treatments. Three were kept anaerobic, three were aerated for 706 days after an initial anaerobic phase and three were aerated for 244 days in between two anaerobic phases. In total, 36 solid and 36 leachate samples were taken. Biolog® EcoPlates™ were used to assess the functional diversity of the microbial community. It was possible to directly relate the functional diversity to the biodegradability of MSW (municipal solid waste), measured as RI_4_ (respiration index after 4 days). The differences between the treatments in RI_4_ as well as in carbon and polymer degradation potential were small. Initially, a RI_4_ of about 6.5 to 8 mg O_2_ kg^−1^ DW was reduced to less than 1 mg O_2_ kg^−1^ DW within 114 days of treatment. After the termination of aeration, an increase 3 mg O_2_ kg^−1^ DW was observed. By calculating the integral of the Gompertz equation based on spline interpolation of the Biolog® EcoPlates™ results after 96 h two substrate groups mainly contributing to the biodegradability were identified: carbohydrates and polymers. The microbial activity of the respective microbial consortium could thus be related to the biodegradability with a multilinear regression model.

## Introduction

Gaseous and liquid landfill emissions pose a significant threat to human health and to the environment. Landfilling strongly affects its surrounding by causing gaseous and liquid emissions (Kylefors et al. 2003). These emissions are driven by biological degradation processes of organic matter present in the landfilled waste (El-Fadel et al. 1997). The biodegradation processes in landfills depend on several factors, including the organic matter content, the landfill age, temperature, oxygen situation, heterogeneity of the deposition and the biodegradability of the organic matter. The latter is of importance also for the management of landfills, as the biodegradability of the waste is directly coupled with the methane generation potential (Komilis et al. 1999).

Globally, in landfills containing untreated municipal solid waste (MSW), organic wastes form the main source for biodegradable carbon. Other potential carbon sources are plastics, cardboard and paper. Especially under anaerobic conditions these materials decay rather slowly. During degradation, the C:N ratio tends to narrow down, as easily degradable carbon is mainly converted to methane (CH_4_) and carbon dioxide (CO_2_). At the same time both, the carbon content and biodegradability of the carbon decrease and more recalcitrant material remains behind.

In recent years, for reducing landfill emissions the so-called bioreactor approach gained traction (Valencia et al. 2009), where it is the goal to reduce long-term emissions of landfills by accelerating the biological degradation of waste material. Landfill in-situ aeration is a *bioreactor*-technique used to mitigate hazardous landfill emissions by introducing oxygen into the landfill body. This leads to a reduction of two harmful emissions: gaseous CH_4_ emissions and ammonia (NH_4_) emissions via leachate. For predicting the remaining emission potential of landfills and determining an end point for in-situ measures, the importance of carbon quality with regard to its biodegradability has been emphasized (Prantl et al. 2006; Brandstätter et al. 2015a).

Currently, the forecasting of landfill emissions is typically based on a first-order decay model (Tabasaran and Rettenberger 1987) that was calibrated under laboratory conditions. This model is widely used by practitioners because the parameter assessment of more complex models is not practically feasible (Majdinasab et al. 2017). A deeper understanding of the underlying degradation process might contribute to improve the forecasting, both for anaerobic and aerobic landfill conditions. The degradation process of organic waste material also plays a pivotal role in composting of organic waste.

A detailed view on the changing carbon quality during microbial waste degradation facilitates the understanding of the process on a biochemical level, thus contributing to a better understanding for landfill emission models as well as for estimating the aeration success. To this end, we applied the method of Biolog® EcoPlates™ to solid waste material under different levels of oxygenation in a 2-year laboratory experiment.

The application of Biolog® EcoPlates™ rarely is considered in the context of solid waste. Examples are applications for sewage sludge analysis (Gryta et al. 2014) or, more recent, for analysis of composting microbial diversity (Zeng et al. 2018) or in lysimeter experiments (Dabrowska et al. 2019). Typical ways for Biolog® EcoPlates™ analysis are the calculation of the AWCD (average well color development) or ecological parameters, such as functional diversity (Garland et al. 2001). However, aggregating the results of Biolog® EcoPlates™ data activity data leads to a significant information loss. With the prevailing work we target to extract more meaningful information from the Biolog® EcoPlates™ method to directly link the microbial physiological diversity to the degradation process. For assessing biodegradability, we analyzed the respiration index after 4 days (RI_4_).

## Materials and methods

### Landfill material

The waste material originated from a compartment of the Rautenweg landfill (48.26° N, 16.48° W) near Vienna (Austria) which was filled in the 1980s and the material was sampled (July 2017) during the drilling of new gas wells and then sieved to ≤ 20 mm. The waste material was collected and on site placed into 200 l drums and sealed. The material was stored under anaerobic conditions and then prior to the installation into the reactors piled and mixed with shovels. It consisted of a typical mix of landfilled waste, showing similar characteristics as the material used in Brandstätter et al. (2015a).

### Experimental setup

The experiment involved the operation of nine landfill simulation reactors, similarly designed as those described in Brandstätter et al. (2015a). The reactors were made of PP (polypropylene) and regularly watered from above. Roughly 10 cm above the vessel bottom, a crate was installed to prevent the waste material from soaking. The total volume of each reactor was 60 l - corresponding to an initial waste material mass of 38.3 kg ± 1.2 kg. More details on the reactors are presented in the work of Fricko et al. (2021).

After an initial anaerobic phase of 57 days, six reactors were aerated—three of them for the complete remaining period of 706 days (aerobic treatment), three others only for 244 days (mixed treatment) and three remained without aeration for the whole period (anaerobic treatment). An Argon-Oxygen mixture (79% Ar (Argon 5.0), 21% O_2_, Messer, Austria) was used as aeration gas. The gas influx into each reactor was recorded with a thermal massflow meter (FMA3103, OMEGA Engineering, Germany) for flow rates between 0 and 0.1 Nl/min and adjusted manually. The temperature of each reactor was recorded and individually controlled (set value 35 °C).

Solid samples were retrieved from each reactor at start and end of the whole experiment. To keep anaerobic conditions intact, no sampling occurred during anaerobic operation periods. The aerated (aerobic and mixed) reactors were sampled five times in total. The conditions during sampling (aeration status) is shown in the brackets before the sample size. The samplings were linked to following events due to changes in operation:

- initial sampling upon installation (also for anaerobic reactors; anaerobic n = 9)
- after an initial anaerobic phase, before start of aeration (57 days; still anaerobic conditions; anaerobic n = 6)
- after two months of aeration (114 days, aerobic n = 6)
- after one year of operation (358 days, aerobic n = 6), directly before terminating aeration for the mixed treatment
- at the termination time point, together with the anaerobic treatment (763 days, anaerobic n = 6, aerobic n = 3)

For the first four solid sampling campaigns (until day 358), the conditions prior to sampling were the same for both the aerobic and mixed treatment (n = 6). At the first sampling campaign upon installation, all the reactors were at the same (anaerobic) state (n = 9). Only at the final sampling campaign after 763 days, at the termination of the experiment, the different treatments were reflected in three solid samples per treatment.

During sampling, the material was intensively mixed, subsequently randomly sampled and sieved (≤ 4 mm). Only this fraction was used for the ecophysiological profiling. In general, leachate was sampled regularly every four weeks with intensified intervals after changes in operation (2 weeks). However, the focus was put on the changes in the microbial community composition over time and differences or intersections between the solid and liquid phase microbiota. Hence, only leachate related to the sampling events was considered for the ecological phenotype microarrays analysis (in total also 36 samples). The resulting solid and liquid samples were processed as fast as possible. Nevertheless, in most of the cases cooled storage (6 °C) was required. Unfortunately in one case (sampling campaign 3, day 114), the samples had to be frozen to −20 °C prior to the processing of the ecological phenotype microarrays.

### Measurement methods

For conducting dynamic monitoring of the functional diversity of the respective samples over six days the Biolog® EcoPlates™ method was used. Every EcoPlate consisted of 96 wells containing 31 carbon sources (see Table 1) plus a blank well in three replications each. The carbon utilization rate was determined by reducing a tetrazolium violet redox dye, which changed from colorless to purple if the microorganisms used the respective carbon source. More technically, the coloring is not directly caused by the substrate usage, but through respiration. For coloring, the microbes must be able to grow/breathe in the respective medium containing single carbon sources. For the analysis, the Biolog® EcoPlates™ substrates were subdivided into five groups (see Table 1): amines, amino acids, carbohydrates, carboxylic acids and polymers (Gryta et al. 2014; University of Toledo 2004). For the respective calculation of the utilization rate, the measured O.D.-values at 590 nm for each substrate, used as an indicator of microbial respiration due to dye color change (Mills and Garland 2002; Pinzari et al. 2017), were summed up to the according substrate groups.

The Biolog® EcoPlates™ to be tested were prepared according to an extraction protocol modified from Hopkins et al. (1991), separating bacterial cells from soil particles. In brief, for the solid samples, 5 g of material was placed in a 250 ml flask containing 50 ml 10 mM sterile phosphate buffer (pH 7.0) supplemented with 0.1% tween 20 (PBS+TW20) and 30 glass beads. For the liquid samples (leachate), all the liquids were centrifuged at 10,000 r min^−1^ for 10 min, and the resulted pellets were washed three times with sterile ultrapure water.

Using a spectrophotometer, the turbidity of each bacterial suspensions’ samples was adjusted to 0.5 McFarland standard turbidity (0.1 O.D. at 595 nm wavelength). This turbidity is equal to approximately 10^6^ of bacterial cells in 1 ml of the samples. For the analysis, the plates were placed into a plastic container and incubated at 28 °C for 7 days. The absorbance at both 590 and 750 nm was measured on a Biolog Microplate Reader (molecular devices) after 24, 48, 72, 96 and 144 h of incubation.

The respiration index after 4 days (RI_4_) was measured according to the Austrian standard ON S 2027-4:2012-06-01.

**Table 1:** Substrates

Substrate group	Substrate
Amines	Phenylethylamine
	Putrescine
Amino acids	Glycyl-l-glutamic acid
	l-Arginine
	l-Asparagine
	lPhenylalanine
	l-Serine
	l-Threonine
Carbohydrates	α-d-Lactose
	d-Cellobiose
	d-galactonic acid γ-Lactone
	d-Mannitol
	d-Xylose
	d,l-α-Glycerol phosphate
	Glucose-1-phosphate
	i-Erythritol
	*N*-acetyl-d-glucosamine
	β-Methyl-d-Glucoside
Carboxylic acids	2-Hydroxy benzoic acid
	4-Hydroxy benzoic acid
	d-Galacturonic acid
	d-Glucosaminic acid
	d-Malic acid
	γ-Hydroxybutyric acid
	Itaconic acid
	Pyruvic acid methyl ester
	α-Ketobutyric acid
Polymers	α-Cyclodextrin
	Glycogen
	Tween 40
	Tween 80

### Data preparation and statistical procedures

All statistical analyses as well as data preparation were performed using R version 3.6.3 (R Core Team 2018). Prior to further calculations, the average blank values were subtracted and then the average measurement value of the technical replicates were calculates (three replicates per well). If certain measurement values were negative after the blank subtraction, they were set to 0. Concerning the handling of missing values for the solid samples, one out of three data points of the mixed treatment at 763 days was missing; for this missing replicate at each measurement the mean of the other two measurements was applied. Missing data points of the leachate samples were not treated (they remained excluded). For some samples, which were measured longer than 96 h, the measurement values were interpolated (spline-interpolation) and cut off at 96 h. For calculating the total substrate consumption a Gompertz equation was fitted by using the grofit package (Kahm et al. 2010). The Grofit-equation commonly used Zwietering et al. (1990) is derived as follows:

1 $$\begin{aligned} y = A \exp {\left\{ -\exp {\left[ \frac{\mu e}{A} (\lambda -t) + 1 \right] }\right\} } \end{aligned}$$

with A being the asymptote (amplitude), μ being the linear slope (or growth rate) and $\lambda$ the lag time. Prior to the calculation of the Gompertz-curve (Eq. 1), spline interpolation was conducted. For the interpolation, the total time period of 96 h was divided in 100 data points based on the measured data points. Based on that interpolation, a heuristic algorithm was applied to fit the best Gompertz equation. In those cases, where no Gompertz equation could be calculated (where the $\lambda$-value was negative), the spline interpolation was considered as base for integration over 96 h. This was also the case, when O.D. was 0 over the whole measurement. More detailed information on the application of the Gompertz calculation is given in Table 2. A successful Gompertz equation means in this context, that the numeric conditions for fitting the Gompertz equation were given and a Gompertz equation could be derived. As the Gompertz curve was fitted on the integral of the spline function, the difference of the integral between those two was considered negligible.

**Fig. 1:**
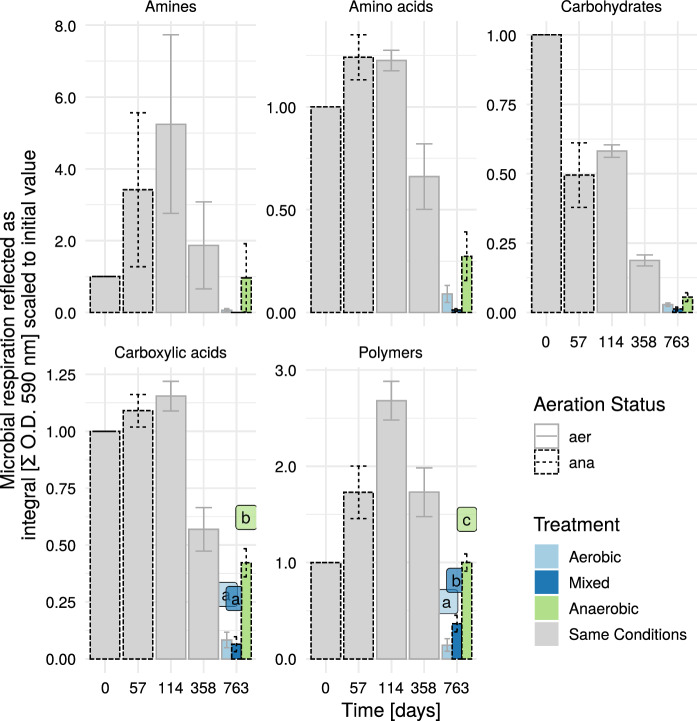
Dynamics of microbial respiratory activity in solid samples depending on the biochemical group of the carbon source and the landfill type. Mixed treatment started anaerobically, then was switched to aerobic (1 year), then to anaerobic again. Different letters indicate significant differences according to Tukey HSD-test (p = 0.05). At the beginning and the end all reactors (n = 9) were sampled. From day 57 to 358 only aerobic reactors were sampled (n = 6). Values were scaled according to values from T = 0. Error bars indicate standard error (day 0: n = 9; day 763: n = 3; all others: n = 6). Aeration status represents the conditions during sampling .*aer* aerobic, *ana* anaerobic, *O.D.* optical density

**Fig. 2:**
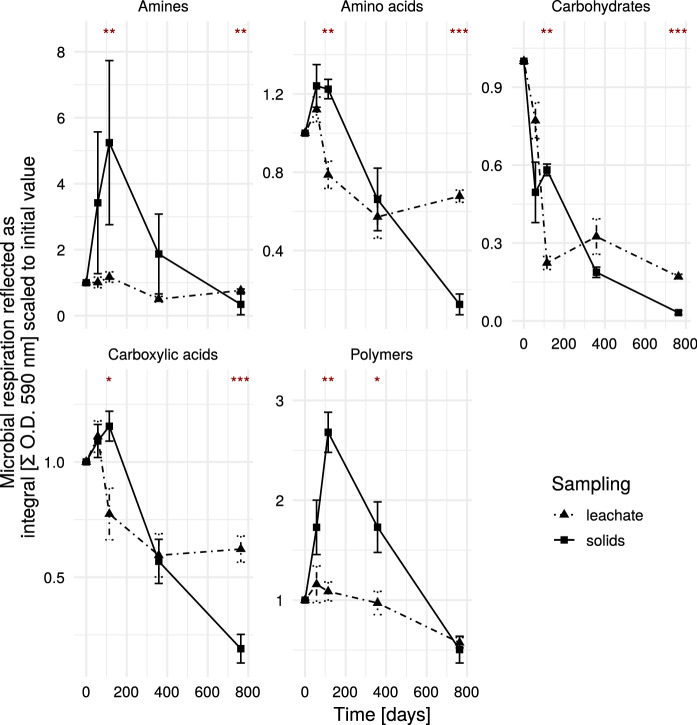
Influence of the sampling method on the microbial respiratory activity—Solids vs. Leachate. The leachate samples directly stem from the reactor leachate, while the solid samples were derived through the elution of solid sample material. Stars indicate significant differences according to Wilcoxon-range-test. At the beginning and the end all reactors were sampled (n = 9). From day 57 to 358 only aerobic reactors were sampled (n = 6). Values were scaled according to values from T = 0. Error bars indicate standard error. *O.D.* optical density. ***$p<0.001$; **$p<0.01$; *$p<0.05$.

For a more meaningful display of the temporal development of the respective substrate utilization throughout the experiment, scaling was applied (see Figs. 1, 2, 5 and 6). Thereto, the initial values (for the integrated substrate utilization) of the respective reactors were referred to the value of the first sampling (day 0). Hence, the respective initial value was set to 1 for each variable and reactor.

For the data of the last sampling of the experiment (day 763), ANOVA followed by TukeyHSD-test was conducted between the three treatments (see Fig. 1) in order to check for statistical differences between the treatments. Prior to ANOVA Levene’s test for homogeneity of variances as well as Shapiro test for normality were conducted to check for ANOVA requirements. With the exception of amines (where no significant differences were detected), the requirements for ANOVA were not violated. For the comparison of groups at different time points, Wilcoxon-range-test was applied (see Fig. 2), as not all subgroups were normally distributed.

Before calculating a multilinear model to predict the RI_4_ (see Fig. 4, all values for each variable were normalized as follows:

2 $$\begin{aligned} x_{norm} = \frac{x - {\rm{min}}(x)}{{\rm{max}}(x) - {\rm{min}}(x)} \end{aligned}$$

## Results

### Application of the Gompertz equation

Even if the successful application of the Gompertz application showed some variety over both time course and treatment, in total out of 1,085 measured curves, the Gompertz-fitting could be applied for 816 times, leading to a success rate of 75% (see Table 2). For further analyses, the integral over 96 h was considered. The values of the Gompertz-integral and the spline-integral showed a high degree of similarity, as spline interpolation was also applied for the Gompertz-fitting. For those cases, where the Gompertz-fitting failed, the integration of the spline interpolation over 96 h was considered.

**Table 2:** Gompertz calculation success rate

Treatment/days	0	57	114	358	763
Aerobic	69/93	54/93	88/93	67/93	72/93
%	(74.2)	(58.1)	(94.6)	(72)	(77.4)
Mixed	76/93	88/93	92/93	52/93	27/62*
%	(81.7)	(94.6)	(98.9)	(55.9)	(43.5)
Anaerobic	87 / 93				44 / 93
%	(93.5)				(47.3)

Left number: successful gompertz equation derivation. right number: total number of substrates in subgroup. number in brackets: Percentage. *In this subgroup one sample was missing—the mean was applied for each measurement. Mixed Treatment starts with anaerobic, then was switched to aerobic (1 year), then anaerobic again

### Microbial respiratory activity on different substrate groups

The different substrate groups showed different responses over the experimental time course (see Fig. 1).

On overall, the microbial metabolic activity and growth of the microbial consortium was lowest at the end of the experiment. This was to be expected in a batch reactor experiment: the more recalcitrant substrate fractions accumulate and easier degradable fractions would become less and less common. At the last sampling campaign, the anaerobic treatment showed higher activity in all substrate groups compared to the aerobic one.

The microbial growth on amines generally was high in variation and showed the highest values in the midterm of the experiment (5-fold increase in comparison to the initial value). The microbial community was able to show highest activity on amino acids in the first half of the experiment. However, increases in activity on amino acids (referred to the initial value) were moderate in comparison to amines. Overall growth/activity on carbohydrates was highest initially and reduced over time to less than 5% of the initial value. For the carboxylic acids, there was a slight increase in the first half of the experiment followed by a decrease. Polymers showed a similar pattern to amines, with a pronounced increase until day 114 and from there onwards decreasing. Significant differences among the three treatment groups (aerobic, anaerobic and mixed) at the final sampling campaign were only found for carboxylic acids and for polymers, with the anaerobic treatment showing the highest utilization capability.

### Sampling strategies

Comparing different sampling strategies (solid and liquid samples), there were significant differences to be found among all substrate groups (see Fig. 2). For the amines, the solids showed an initial stronger increase than the liquids. Apparently, at the sampling campaign of day 114, the solid samples showed higher relative values (compared in to the initial one), while at the last sampling campaign, this trend was inverse, with leachate samples showing higher values. Also the variation at day 114 was relatively high. Both sampling strategies revealed similar trends over time.

### Relationship between the microbial activity and biodegradability

For measuring the biodegradability of municipal solid or organic waste, the RI_4_ is often applied as a proxy-measure due to its reproducibility and standardization (Binner et al. 2012; Binner and Zach 1999). In waste management, knowledge of the biological degradability of the waste material is important, as it is a driving factor of future emissions. In Austria and Germany, for waste material a low RI_4_ is necessary prior to landfilling (for Austrian landfills, the threshold value was set to 7 mg O_2_ kg^−1^ DW). Generally, respiration indices are used to determine microbial activity of waste samples containing organic fractions and they can be measured via CO_2_ production or O_2_ consumption under standardized conditions (Barrena Gómez et al. 2006). High values of RI_4_ indicate a high potential for microbial respiratory activity.

**Fig. 3:**
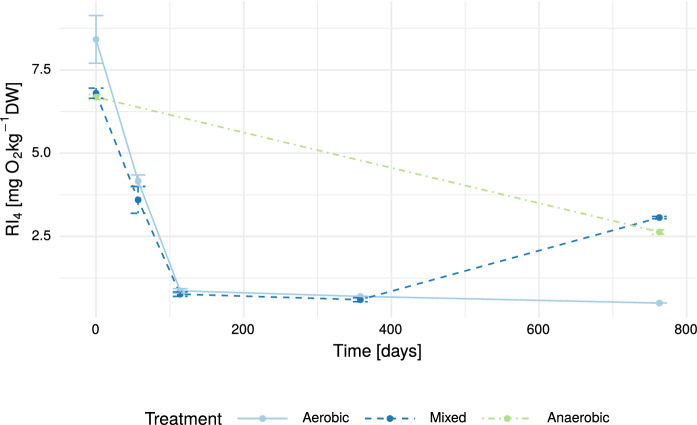
RI_4_ of landfilled waste in each treatment during the experiment

Here, we observed a strong decrease for the aerobic and mixed treatment in the beginning from about 6.5 to 8 mg O_2_ kg^−1^ DW to less than 1 mg O_2_ kg^−1^ DW within 114 days of treatment (Fig. 3). There also was a decrease in the anaerobic case observable, but less pronounced over time (from 6.5 to 2.6 mg O_2_ kg^−1^ DW after 763 days of treatment). A notable increase of RI_4_ in the mixed treatment after aeration stop to 3 mg O_2_ kg^−1^ DW is surprisingly even higher than the RI_4_ observed for the anaerobic treatment at the end of the experiment.

**Fig. 4:**
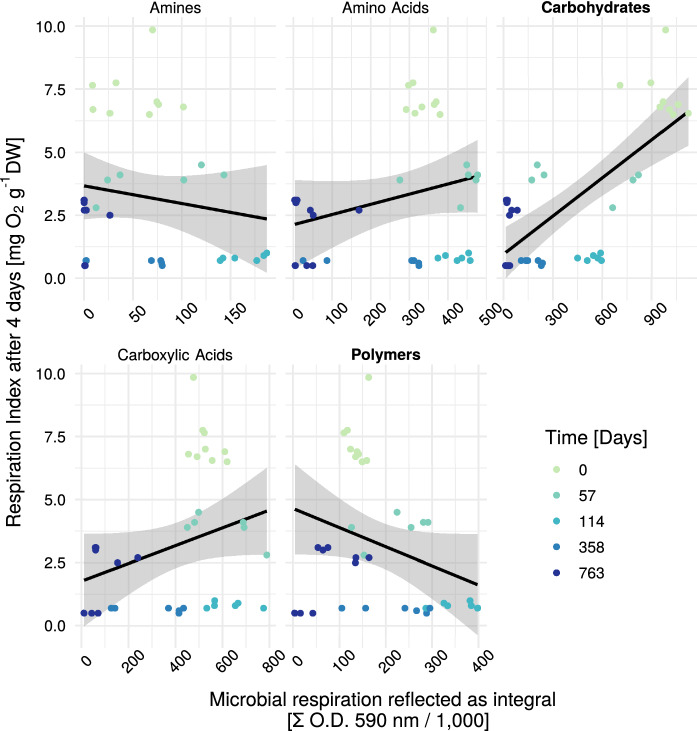
RI_4_ vs Substrate group. The shaded area shows the CI at the 95% level. *O.D.* optical density

Since the biodegradability of municipal solid waste is of high interest for landfill practitioners as well as for the assessment of their environmental impact, an increased understanding of its driving forces is important. The relationship between the degradation capability of the microbial consortium and the RI_4_ is displayed in Fig. 4. A strong linear relationship can be observed between Carbohydrates and RI_4_, and a weaker negative relationship between RI_4_ and polymer degradation. The relationship between amines, amino acids and carboxylic acids with RI_4_ is less pronounced. Thus, we considered the carbohydrates and polymers for a multilinear model to predict RI_4_. The resulting model can be described as follows:

3 $$\begin{aligned} RI_4 = 0.26 + 0.68*Carbohydrates - 0.5*Polymers \end{aligned}$$

with Carbohydrates and Polymers being the standardized integral of the of the O.D. at 590 nm for all parameters of its subgroup (see Table 1). All the model parameters (including intercept) showed a p-value < 0.001 and the adjusted R^2^ value was 0.69 based on 36 samples.

**Fig. 5:**
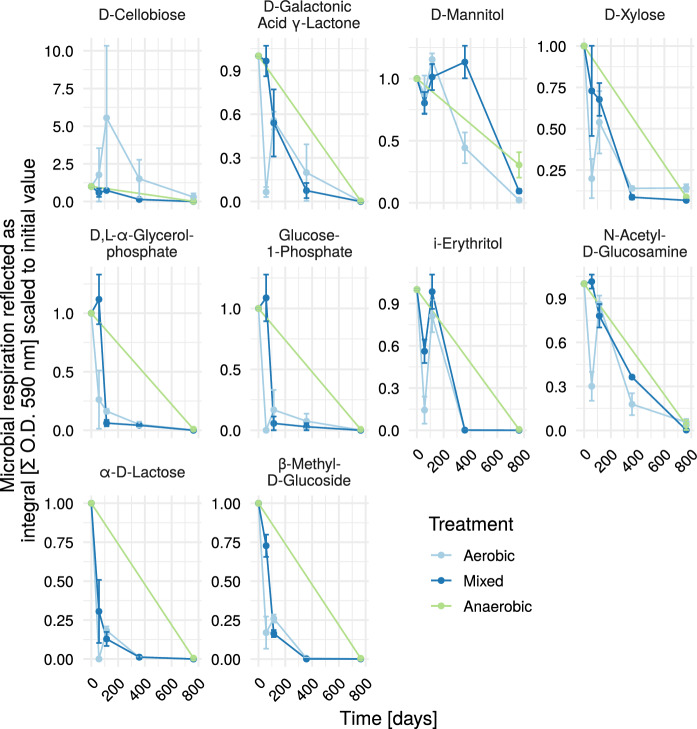
Timeline carbohydrates. Mixed treatment started anaerobically, then was switched to aerobic (1 year), then to anaerobic again. At the beginning all reactors were sampled. From day 57 to 358 only aerobic reactors were sampled. Values were scaled according to values from T = 0. *O.D.* optical density

**Fig. 6:**
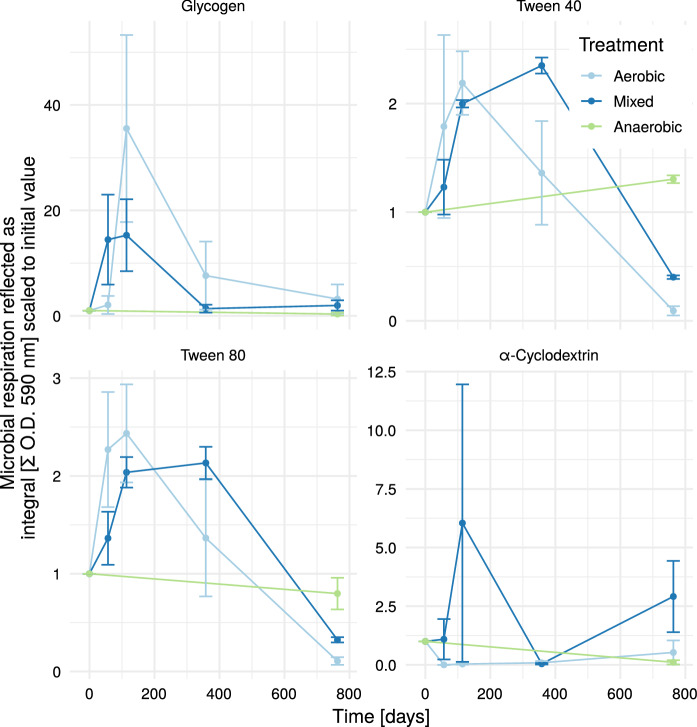
Timeline polymers. Mixed treatment started anaerobically, then was switched to aerobic (1 year), then to anaerobic again. At the beginning all reactors were sampled. From day 57 to 358 only aerobic reactors were sampled. Values were scaled according to values from T = 0. *O.D.* optical density

Since these two substrate groups are highly associated with short-term biodegradability of municipal solid waste, the individual components are shown in Figs. 5 and 6. Similarly to the RI_4_ (Fig. 3), the individual carbohydrate fractions (Fig. 5) show a rapid decrease early in the experiment. This was the case except for D-cellobiose, the monomer of cellulose, where the decrease was slower and for i-erythritol, where there was a peak at 114 days. The treatment did not show a big impact on the capability of the microbial community to utilize different carbon sources at the end of the experiment.

For the polymers (Fig. 6), the time course of the degradation capability showed an inconsistent picture. Both tween-fractions showed an initial increase followed by a strong decrease for the aerated treatments and a slight change in the anaerobic treatment. The time course of α-cyclodextrin revealed an increase for the aerated treatments with high variation in between and glycogen showed an increase until day 114 and from there a decrease. The degradation capability for glycogen was highest for the (strictly) aerobic treatment. Glycogen is an important storage molecule for numerous microorganisms. Its buildup or decomposition is controlled by environmental factors, such as glucose-concentration (Wilson et al. 2010) and in comparison to the other polymers in the substrate group it is less recalcitrant.

## Discussion

### Validation of methodological approach

The common usage of average well color development (AWCD) may lead to a loss of the ecological signature of a sample (Miki et al. 2018). It was noted by the authors, that the integration of the signal showed a higher statistical power than using other approaches, like min or max values. Like others (Sofo and Ricciuti 2019), we observed that the detailed description of the usage of Biolog® EcoPlates™ data is often lacking. It is the aim of this work, to increase the level of standardization of the method. This is not only important for natural soils, but also of high relevance in solid landfill samples, showing already highly heterogeneous properties. For the paper at hand, it was not possible to calculate the AWCD, as the measurements at T = 0 were not performed. This also might have lead to a slight signal underestimation in the chosen approach of integrating a fitted curve, since T = 0 was considered as 0. For future approaches, it is important to also measure at the initial setup.

The main motivation behind using the Gompertz equation was that it describes a natural process, namely biological growth. This makes the interpretation more meaningful, instead of directly using the measured values for integration. The results showed, at least for carbohydrates and polymers, surprisingly low variation between the treatments as well as between time points (see Figs. 5 and 6). For amino acids and amines the variation was much higher (data not shown). This might be explained by the composition of old landfilled waste. The total carbon content of the material is 5- to 10-fold higher than the total nitrogen concentration (Brandstätter et al. 2015a, b). Thus, more abundant carbon fractions show lower error values than less abundant nitrogen-fractions.

Out of those samples/substrates, where a Gompertz-fitting could not be performed (n = 269), 116 showed a integrated value lower than 96 (1 O.D./h). This shows, that a big fraction of samples, where the Gompertz equation could not be fitted, were rather low in respiration activity. For those measured entities, where both spline interpolation and Gompertz-fitting were applied, the correlation coefficient between those two was 0.999 (data not shown). This is a direct result of the fitting method, as the Gompertz fitting was based on the spline function.

### Decomposition of individual substrate groups: experimental influence

The initial sampling occurred during the reactor construction. The waste material was kept for roughly two years at room temperature under anaerobic conditions. Before sampling at the first time point, the material got thoroughly mixed and during this procedure also oxygenated to some extent. Thus, we created an ecological disturbance event of mostly (strictly and facultative) anaerobic microorganisms. This could have affected the high utilization rates of carbohydrates (see Fig. 1) at the very early stage of the experiment. For the substrate group carbohydrates, nearly all of the individual carbohydrate substrates showed initially a very high utilization rate, followed by a strong decrease (see Fig. 5). Other reasons for this initial high peak might be an accumulation of carbohydrates during this 2 years of anaerobic incubation, that were not accessible under anaerobic conditions. With the mixing and oxygenation there presumably was a sudden abundance of readily available carbohydrates, also possibly impacted by the death of strictly anaerobic microorganisms.

During the degradation experiment, after the initial anaerobic setup with leachate recirculation and heating, at one point oxygen got introduced to the anaerobic system for the mixed and aerobic treatment. This lead to another ecological disturbance and the introduction of oxygen allowed for previously inaccessible microbial substrate utilization. Through aeration, the microbial CO_2_ respiration rate drastically increased as it was the case in similar experiments (Brandstätter et al. 2015a; Prantl et al. 2006). Thus, the microbial consortium had to adapt to a richer environment. For most substrate groups (except for carbohydrates and, by a small margin, amino acids), the measured activity was highest at time point three (at 114 days), the first sample after aeration start (and after roughly 2 months of aeration).

At the end of the experiment (day 763) the higher utilization rates of the anaerobic treatment in comparison to the other two treatments for all substrate groups might have been caused by two effects: firstly, the aerated samples were more biologically stabilized and thus resource depleted. Thus the microbiological consortium adapted to a more scarce environment. And secondly, similarly to the initial setup, the sudden oxygenation of previously anaerobic conditions (and the substrate mixing) might have created access to previously not accessible resources.

The experimental design did not allow for in-between sampling of the anaerobic treatment. Thus, for the anaerobic treatment only one sample campaign could be conducted after 2 years. A direct comparison with the other treatments (aerated and mixed) therefore is difficult. The anaerobic treatment was considered as a default case for comparing the in-situ aeration treatment with traditional landfilling, as was done in previous studies (e. g. Prantl et al. 2006; Ritzkowski and Stegmann 2013). For a more direct comparison between the anaerobic and aerobic cases, more anaerobic reactors could be included and destroyed at different time points. However, since the experiment targets to simulate to some extend landfill behavior, landfill simulation reactors should not be too small, making their creation and operation rather costly. Other ways to gain samples from such anaerobic reactors and retain anaerobic conditions would be either a complete anaerobic chamber (also not really feasible) or solid sampling entrance points directly integrated in the reactors (but this would not allow for sample homogenization). In the here presented setup, potential anaerobic effects strongly were influenced by batch reactor resource depletion because of the long time span of 763 days between the initial and the final sampling.

### Sampling strategies

Solid sampling of landfills is often costly and the heterogeneity of the landfilled material heavily influences the results of the analysis (Sormunen et al. 2008; Östman et al. 2006). Thus, it is more common to analyze landfill leachate samples, which are considered to give a better representation of the overall properties of the landfill body. However, through preferential flow paths the leachate is not passing the whole landfill in a similar way (e.g. Huber et al. 2004), and until the leachate sample is collected, it may remain some time in the leachate tank and/or change its properties (e.g. temperature, oxygen) until collection and analysis. In the prevailing case of landfill reactors, preferential flow paths are less pronounced than in the field, as the included material was sieved and mixed. Also the irrigation system was set up to ensure a rather uniform water distribution. During the sampling campaigns there was no evidence of dry patches in the material, which would be a clear sign of heterogeneous water flow. As can be seen in Fig. 2, the variation for both sampling strategies (solid and liquid sampling) was generally of a similar magnitude. For the solid samples, there was visibly higher variation in amines and amino acids and polymers in comparison to the leachate samples, for carbohydrates the opposite was the case.

The amines showed a rather high variation in the solid samples. One reason for this might be that this substrate group contained the minimal number of substrates: two (phenylethylamine and putrescine). The dominant pathway for the biological production of amines is the decarboxylation of amino acids (Halász et al. 1994). Influenced by the before mentioned heterogeneity of the waste material, a low occurrence of the raw material (amino acid concentrations, data not shown) might be a driving force in altering the ability of the microbial consortium for amine consumption. Amino acids are a valuable resource for many cellular activities and other uses might have been preferred over amine production, thus reducing the ability of the microbial community for amine consumption.

At the final sampling point, the microbial degradation potential was generally lower for the solid samples. This might be attributed to a few reasons: first the leachate is getting a more integrated sample of the overall community, and the solid samples are more prone to sampling biases. Second, it might be the case, that in the leachate at the bottom of the reactors a more diverse microbial flora emerged, thus enriching the leachate sample in microbial diversity. In a similar experiment, denitrification was considered to mainly occur in externalized leachate tanks (Brandstätter et al. 2015b), as these leachate reservoirs got charged with carbohydrates from leachate recirculation and provided anaerobic conditions.

Furthermore, bacteria tend to form biofilms and attach to solids (e. g. Cai et al. 2019). Therefore the analysis of leachate does not address the complete biological functionality present in the samples. In this study, we attempted the extraction of microbial cells from solid particles and considered the samples from the solids as more trustworthy, as the solid dataset was more complete and the samples would better represent real landfill conditions. We generally ensured a profound mixing procedure to reduce variability as much as possible.

### Insights on biodegradability

The here investigated system contains landfill bioreactors under different oxygen conditions. This means, that next to classical anaerobic biodegradation of landfilled waste, also the aerobic degradation was investigated. In waste management, the composting process investigates aerobic degradation of organic waste materials. There, substrate quality is of high relevance for the process assessment (Komilis 2015; Meng et al. 2019). Compost stability was technically defined as a measure of the resistance against further microbial decomposition. During the degradation of organic matter in a batch system, the material naturally gets more and more recalcitrant, as readily degradable substances are getting oxygenated into CO_2_ or, in the anaerobic case, also reduced to CH_4_. More recalcitrant components of MSW are wood and rubber (Patil et al. 2017). These are characterized by bigger and more complex compounds, which are represented in the substrate group polymers (see Figs. 4 and 6 ). The here investigated waste material was taken from a rather old landfill, thus it is not surprising, that polymer degradation was found to be a relevant predictor for the respiration index.

The RI_4_ is of high relevance for authorities to determine waste reactivity (Binner et al. 2012). As it describes the oxygen consumption of a subsample of material under a rather short amount of time, the influence of rather readily degradable carbohydrates is considered plausible (see Fig. 5). The differences between the treatments in RI_4_ as well as in carbon and polymer degradation potential were surprisingly small. A conclusion from that could be, that for this old waste material, the laboratory conditions, namely mixing, irrigation and heating were of higher relevance for the formation of the microbial consortia than the differences in oxygen addition.

To determine an end-point for the remediation technique landfill in-situ aeration proves rather challenging (Ritzkowski and Stegmann 2012; Brandstätter et al. 2020). It is known, that the waste stability is increased right after the aeration treatment (Prantl et al. 2006; Ritzkowski and Stegmann 2013). But what happens after the aeration is terminated and the landfill would fall anaerobic again? This is especially interesting for the most relevant nitrogen species in landfills (NH_4_), but here we found in the laboratory an increase in RI_4_ one year after the termination of aeration (last sampling mixed treatment, see 3). We hypothesize, that with the loss of oxygen, after a microbial death event of strictly aerobic microbiota, an accumulation of byproducts occur, that are not fully degradable under anaerobic conditions. By oxygenating the anaerobic material during the RI_4_ testing, these stored compounds could then get subjected to aerobic respiration. It is to be investigated further, whether this observed phenomenon is an artifact under test conditions or if it also would pose difficulties in the full-scale application of in-situ aeration after its termination.

## Conclusions

By investigating MSW-waste degradation of old landfilled waste with Biolog® EcoPlates™  , it was possible to link the metabolic activity of the microbial consortium with the reactivity of the material. Namely, the potential for growth and respiration on carbohydrates (positively) and the potential for utilizing polymers (negatively) both impacted the RI_4_.

We also could observe an increase of the RI_4_ 1 year after the termination of aeration. This needs to be investigated further, as under field conditions, uncontrolled carbon release or punctual temperature increases might occur after the termination of the measures.

## Data Availability

The raw measured data are published on Zenodo (Brandstätter et al. 2021).

## Code availability

In an accompanying master thesis (Brandstätter 2021) the most important R-scripts as well as calculated data are included.
